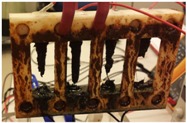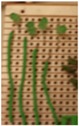# Corrosivity Sensor for Exposed Pipelines Based on Wireless Energy Transfer

**DOI:** 10.3390/s17061238

**Published:** 2017-05-30

**Authors:** Lydia Lawand, Oleg Shiryayev, Khalil Al Handawi, Nader Vahdati, Paul Rostron

**Affiliations:** 1 Department of Mechanical Engineering, Khalifa University of Science and Technology, Petroleum Institute, P.O. Box 2533, Abu Dhabi, UAE; lyslawand@pi.ac.ae (L.L.); KhBAlHandawi@pi.ac.ae (K.A.H.); nvahdati@pi.ac.ae (N.V.); 2 Chemistry Department, Khalifa University of Science and Technology, Petroleum Institute, P.O. Box 2533, Abu Dhabi, UAE; prostron@pi.ac.ae

**Keywords:** corrosion, wireless energy transfer, passive sensors, corrosivity

## Abstract

External corrosion was identified as one of the main causes of pipeline failures worldwide. A solution that addresses the issue of detecting and quantifying corrosivity of environment for application to existing exposed pipelines has been developed. It consists of a sensing array made of an assembly of thin strips of pipeline steel and a circuit that provides a visual sensor reading to the operator. The proposed sensor is passive and does not require a constant power supply. Circuit design was validated through simulations and lab experiments. Accelerated corrosion experiment was conducted to confirm the feasibility of the proposed corrosivity sensor design.

## 1. Introduction

Integrity of pipelines is a serious concern in the oil and gas industry due to potentially catastrophic consequences of pipeline failures. Most pipes used to transfer hydrocarbons in the fields are made of low carbon steel because it is inexpensive and has suitable mechanical properties. Unfortunately, low carbon steel turns out to be very susceptible to corrosive environments, especially oxygenated waters. This significantly affects the lifespan of the equipment. Leaking pipelines can cause environmental pollution and loss of valuable resources. Among other factors, external corrosion is identified as one of the major causes of pipeline failures. The potential consequences of pipeline corrosion failures can be catastrophic and should not be underestimated. According to the statistics reported by the Pipeline and Hazardous Materials Safety Administration (PHMSA), about 9.4% of pipeline damage incidents between the years 1994 and 2013 were caused by external corrosion [1]. Supporting statistics are also available for Europe. The 8th European Gas Pipeline Incident Data Group (EGIDG) report has revealed that 16.1% of gas pipeline incidents in the period from 2007 to 2010 were caused by corrosion problems [2].

The main objective of this research project is to create a sensing solution for monitoring corrosivity of the environment in the vicinity of existing exposed pipelines that could be accepted for use by the oil and gas industry. This sensing solution will facilitate development of so-called “corrosion maps” that indicate locations along the pipeline that are prone to corrosion. We are particularly concerned with the smaller 4–8 inch diameter flow lines that are not inspected regularly, but for which it is necessary to obtain some idea about the range of possible corrosion rates as a function of location along the pipe. We envision that these sensors could be deployed at the intervals of 10–20 m along the pipe to provide sufficiently high spatial resolution for a typical gathering pipeline that is about 2–4 km long.

The purpose of corrosivity sensor is to evaluate the large scale corrosion activity due to exposure in the outdoor atmosphere. It is not possible to make an accurate (within a few percent) estimation of corrosion rate on a specific location on the pipe (e.g., 0.1 m long segment) based on the output of such sensor. The sensor will rather help to classify the corrosivity of the environment to which the pipe is exposed. ISO 9223 [3] standard specifies five corrosivity classes corresponding to environments with different corrosion rates: industrial, tropical marine, temperate marine, urban, and rural. The data obtained from interrogation of this sensor will allow direct classification of the environment near the sensor to which the pipe is subjected and enable the users to estimate the approximate range of atmospheric corrosion rates. There are several common methods for assessment of atmospheric corrosion [4,5]. Some of these methods rely on monitoring of atmospheric corrosivity factors such as relative humidity, temperature, time of wetness, and industrial pollutants (e.g., SO_2_, NO_*x*_). Others are based on weight loss measurements of coupons exposed to the environment. Application of such techniques to obtain a corrosion map of a specific pipeline will be prohibitive in terms of involved costs and time because it will require deployment of hundreds of sensors measuring corrosivity factors, or installing hundreds of sets of coupons along a given pipeline and performing weight loss measurements for all of them.

The ideal sensing solution must possess the following characteristics in order for it to be widely accepted and implemented by the industry:

- The sensor must not require a continuous power supply. In other words, it should be passive.
- The sensor must not interfere with existing pipe structure. It shall not require stopping transportation of hydrocarbons through the pipe during installation and operation.
- The proposed sensor should be inexpensive considering that a large quantity of sensors will be necessary to instrument any realistic pipeline.
- Installation and replacements costs must be low.

External corrosion of pipelines is an environmental phenomenon fueled by the presence of oxygen, water and a conductive metallic material such as steel used in the pipeline’s construction. External corrosion is a consequence of the difference in electrochemical potential between anodic and cathodic regions that form on the steel pipeline due to the difference in oxygen concentration at either site. Anodic sites are susceptible to oxidation of the iron atoms resulting in thickness loss [6].

Large diameter oil transmission pipelines can be inspected to assess the degree of corrosion that has taken place. Pulsed Eddy Current (PEC) [7], radiography techniques [8], Fiber Bragg Grating (FBG) [9] hoop-strain sensors, and Magnetic Flux Leakage (MFL) [10], transmission ultrasonic and long-range ultrasonic inspection are well established conventional Non-Destructive Testing (NDT) techniques that assess the structural integrity of components without material removal or permanent damage to the part. However, the above-mentioned techniques consume large amounts of energy for sensor excitation, signal conditioning and communication of the transduced signal. Furthermore, certain techniques are highly sensitive to location and sensor configuration. Other techniques such as the optical methods described by Ref. [9] required compensation techniques to decouple thickness loss measurements from other measurands such as temperature and internal pressure fluctuations, which may obscure hoop strain measurements.

In many cases, a direct assessment of the pipeline condition using existing NDT methods is impractical or even impossible to do, such as, for example, for small diameter flow lines. These lines connect the wells with field processing facilities and are typically four to eight inches in diameter. Hence, an indirect assessment is usually done by inferring corrosion rates from assessing the corrosivity of environment. In this paper, we propose an approach to assess the corrosivity of environment that satisfies the sensing solution characteristics described earlier in this section.

The proposed approach relies on wireless energy transfer as a method for energizing the sensor and communicating its condition to the operator. Wireless energy transfer is being used in various applications in the fields of biotechnology, energy management and Radio Frequency Identification (RFID) devices. Several studies have been done in the past to develop RFID based sensors for corrosion monitoring of structures. A consumable element in the circuit interacts with the environment causing a variation in the inductance of the antenna that is used to communicate signals to the reader circuit. The altered inductance changes the electromagnetic response of the reader circuit allowing it to infer the condition of the consumable element, and hence the corrosivity of its environment [11]. A similar concept has been developed by He et al. [12,13].

Other solutions for aircraft structures have been developed [14]. This design represents a sensor consisting of two RFID chips, an antenna and an intermediate switch that disconnects the antenna in the event a health problem is detected. Wires covered with soluble coats form the switch which, upon complete dissolution of the coats, results in a short-circuit and termination of the antenna connection to the RFID chip. Other passive wireless corrosion sensors [15] include a corrosion sensitive link connecting the circuit to the antenna. When exposed to the surrounding environment, the corrosion sensitive link will get corroded, breaking the circuit, and hence the sensor will not be able to send a response signal back to the transceiver.

Alamin et al. [16,17] devised a sensor system that utilizes commercial RFID tags and readers where the tags are simply attached to the metal surface. Detection of corroded metal is based on the changes in amplitude of the response waveform sent by the tag to the reader. An extension of this work [18] considers changes in conductivity and permeability in the corrosion layer. Leon-Salas et al. [19,20] developed a sophisticated RFID-based sensor based on a microcontroller. The sensor is meant to be embedded into reinforced concrete structures and can be used to make several measurements including temperature, half-cell potential, and linear polarization. Another corrosion sensor for monitoring of reinforced concrete structures was developed by Satoh et al. [21]. This sensor is based on measurements of conductivity in the sensing film.

Wireless transfer devices rely on a consistent data stream between the sensing and interrogator circuits, which in close vicinity to a steel structure such as a pipeline, have their data streams compromised due to electromagnetic interference and shielding effects [15]. To mitigate reliability issues with the data stream, Ref. [22] proposes a passive LC coil resonator that relies on changes in its resonant frequency to interrogate changes in the sensing circuit. The sensing circuit is embedded in the concrete structure and is connected to the reinforcing steel while using a stainless steel reference electrode. Corrosion alters the cell potential of the two electrodes altering the resonant frequency. The technique presented is useful for instrumenting new structures. Retrofitting existing pipelines and structures would prove difficult. Furthermore, interrogating changes in the resonant frequency employs complex circuitry and post-processing of data to infer corrosion damage. It was therefore decided to develop a passive sensor, the reading of which can be easily interpreted by visual observation either by a human operator or via an unmanned vehicle traveling along the pipeline. The next section presents the proposed corrosivity sensor design.

## 2. Sensor Configuration

The proposed sensor has a sensing array that consists of rectangular strips made of the same metal as that of the pipe. All strips have the same planar dimensions but have different thickness. Each strip in the sensing array is connected to a Light Emitting Diode (LED) and constitutes a branch of the sensor circuit. The circuit branches are arranged in parallel. As corrosion propagates in the metal strips, it consumes the metal until it finally breaks the metal strip apart, resulting in a discontinuity in the corresponding branch of the circuit. This effectively disconnects the corresponding LED so that it will not emit light when the sensing circuit receives power via wireless energy transfer from the interrogating transmitter. The thinnest strip is expected to fail first and the thickest one last.

**Figure 1:**
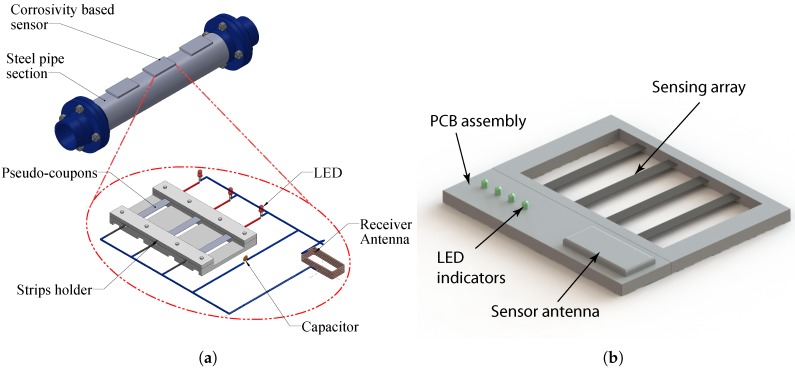
Schematic of the proposed sensor design. (**a**) sensor diagram; (**b**) sensor packaged for field deployment.

Figure 1a shows a schematic of the proposed sensor design. Figure 1b shows the render of the sensor packaged into an enclosure suitable for deployment in the field. The sensor circuit will be realized in the form of the printed circuit board assembly, which will allow for reduction in size of the sensor and reduce manufacturing costs when the sensors are mass produced. The circuit board assembly must be housed inside a sealed enclosure that will protect it from the environment, but the sensing array will be exposed to the atmosphere. The sensors will be placed near the pipeline at reasonable intervals (e.g., 10–20 m). In order to probe the sensors, the inspector will walk along the pipeline, placing the interrogating antenna in immediate vicinity of the sensor antenna and observe the response of the LEDs.

Unlike the RFID-based sensors that exist in the literature, the proposed sensor does not rely on data exchange between the RFID reader device and the sensor tag, hence improving its reliability. The proposed sensor can be used for creation of corrosion maps, and it satisfies most of the requirements desired by the industry.

In order to study the initial feasibility of the proposed sensor design, an initial simplified model of both the sensor and reader circuits were created and tested through lab experiments as shown in our previous work [23]. Good agreement between the results from the experiment and simulation was observed. As expected, the amplitude of the signal transferred to the sensor decreased as the distance between the two antennas increased. This paper presents further investigation to determine the maximum value of resistance for each sensing strip at which its corresponding LED will stop emitting visible light. In addition, we present the result of an accelerated corrosion test of the sensor prototype performed using an electrochemical cell.

### 2.1. Simulation Model of the Proposed Sensor

**Figure 2:**
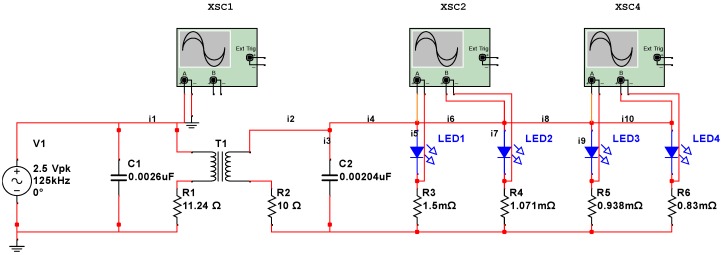
Sensor circuit model.

A model of the sensor circuit was created using NI Multisim circuit modeling software (v. 14.0, National Instruments Corporation, Austin, TX, USA). The circuit consists of four LEDs assembled in parallel branches. Each LED is connected to a resistor in series that represents the electrical resistance of each metal strip. In this work, we considered 125 kHz frequency typically used in low frequency RFID systems. Typical working range of low frequency RFID systems and wireless charging stations is just a few centimeters. Standard characteristics of wireless charging devices can be obtained from specifications listed in Ref. [24]. Figure 2 shows a schematic diagram of the circuit containing four LEDs. The transmitter is shown in the left part of diagram and contains a resonant circuit with a voltage source.

Antennas are represented by equivalent inductors and resistors, with both mutual and self-inductance taken into consideration. Equations (1)–(10) show mathematical representation of the nonlinear circuit with four LEDs that is implemented in this model:

(1) $$\begin{array}{c} \frac{di_{1}}{dt}=\frac{v_{i}}{L_{1}}+\frac{M}{L_{1}}\frac{di_{2}}{dt}-\frac{R_{1}i_{1}}{L_{1}}, \end{array}$$

(2) $$\begin{array}{c} \frac{di_{2}}{dt}=\frac{M}{L_{2}}\frac{di_{1}}{dt}-\frac{R_{2}i_{2}}{L_{2}}-\frac{1}{C_{2}L_{2}}\int i_{3}dt, \end{array}$$

(3) $$\begin{array}{c} v_{1LED}=M\frac{di_{1}}{dt}-L_{2}\frac{di_{2}}{dt}-R_{2}i_{2}-R_{3}i_{5}, \end{array}$$

(4) $$\begin{array}{c} v_{2LED}=M\frac{di_{1}}{dt}-L_{2}\frac{di_{2}}{dt}-R_{2}i_{2}-R_{4}i_{7}, \end{array}$$

(5) $$\begin{array}{c} v_{3LED}=M\frac{di_{1}}{dt}-L_{2}\frac{di_{2}}{dt}-R_{2}i_{2}-R_{5}i_{9}, \end{array}$$

(6) $$\begin{array}{c} v_{3LED}=M\frac{di_{1}}{dt}-L_{2}\frac{di_{2}}{dt}-R_{2}i_{2}-R_{6}i_{10}, \end{array}$$

(7) $$\begin{array}{c} i_{8}+i_{7}=i_{6}, \end{array}$$

(8) $$\begin{array}{c} i_{6}+i_{5}=i_{4}, \end{array}$$

(9) $$\begin{array}{c} i_{4}+i_{3}=i_{2}, \end{array}$$

(10) $$\begin{array}{c} i_{10}+i_{9}=i_{8}, \end{array}$$

where $v_{i}$ is the voltage signal at the voltage source; $i_{1–10}$ are currents across different circuit components as shown in Figure 2; $L_{1}$ and $L_{2}$ are inductance values of transmitter and receiver inductors, respectively; $C_{2}$ is the capacitance; $R_{1}$, $R_{2}$, $R_{3}$, $R_{4}$, $R_{5}$, and $R_{6}$ are resistance values of strips and antenna coils; and *M* is the mutual coupling coefficient that can be calculated using Equation (11) (see Ref. [25]):

(11) $$M=-N_{2}\frac{N_{1}\mu l_{1}l_{2}}{\pi }\left(\frac{1}{\left(l_{2}^{2}+r^{2}\right)\sqrt{l_{1}^{2}+l_{2}^{2}+r^{2}}}+\frac{1}{\left(l_{1}^{2}+r^{2}\right)\sqrt{l_{1}^{2}+l_{2}^{2}+r^{2}}}\right)a_{1}b_{1}.$$

Here, $N_{1}$ is the number of turns in the transmitter coil, $N_{2}$ is the number of turns in the sensor coil, μ is the permeability of free space $\left(\mu =\;4\pi \times 10^{-7}\;N/A^{2}\right)$, $l_{1}$ and $l_{2}$ are the half-length and half-width of the transmitter coil, respectively, $a_{1}$ and $b_{1}$ are the length and width of the sensor coil, respectively, and *r* is the distance between the transmitter and sensor coils.

The coefficient of coupling used in the simulation model was calculated using Equation (12) given by the NI Multisim coupled inductors block, where *M* is the mutual coupling coefficient and $L_{1}$ and $L_{2}$ are the coils’ inductances. By changing the value of coefficient of coupling *K*, we are able to control the read distance between the two inductors (antennas) in the model. The coefficient of coupling value used in the simulation model was K=0.25:

(12) $$K=\frac{M}{\sqrt{L_{1}L_{2}}}.$$

## 3. Model Validation

**Figure 3:**
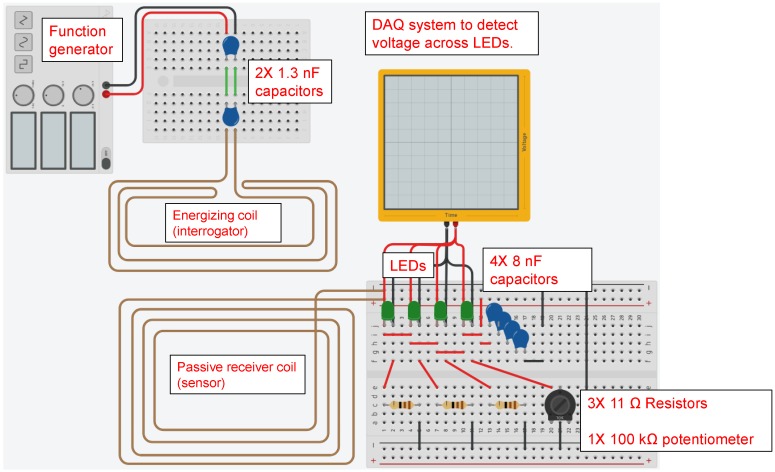
Diagram of experimental circuit for model validation.

A physical sensor circuit represented by the model described in Section 2.1 and shown in Figure 2 was constructed. The diagram of the constructed circuit is shown in Figure 3.

**Figure 4:**
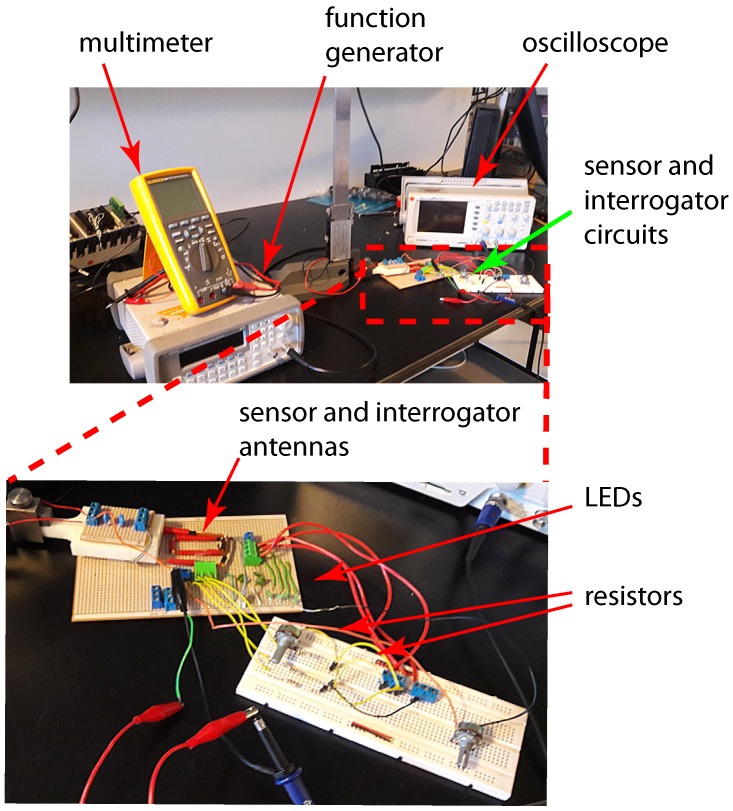
Experimental setup of circuit for model validation.

Figure 4 shows the photos of the experimental setup of the circuit with four LEDs and resistors that is equivalent to the circuit that will be used in the proposed corrosion sensor. The resistors in the circuit represent the sensing strips with different thicknesses. Green (3 mm) diffused LED lamps were used in the experimental setup. The transmitter (reader) and sensor (tag) antennas were mounted directly opposite to each other at a 2 mm distance using a height gage. Excitation voltage signal was generated by a function generator and the signals across each LED in the sensor circuit were logged using an oscilloscope.

### 3.1. Modeling of the Sensing Strips

The sensing strips used in the sensor that we tested in this work had thicknesses of 0.5 mm, 0.7 mm, 0.8 mm, and 0.9 mm. Real corrosion processes are usually fairly slow, and it may take several weeks or months to run a meaningful test in the actual outdoor environment. From a practical stand point and due to time constraints, it was decided to utilize an electrochemical cell in order to run an accelerated corrosion test and be able to control the rate of corrosion. Prior to running the accelerated corrosion experiment, the time required to corrode each strip was estimated using Faraday’s laws of electrolysis shown below:

(13) $$m_{l}=\frac{IT}{F}\frac{M}{z},$$

where I is the current through the electrode, *T* is the time, *F* is Faraday’s constant 96485 C/mole (electric charge per mole of a substance), M is the molar mass of the substance, g/mole, and *z* is the valency number of ions formed (z=2). Here, the current passing through each strip I was 1 A, and $m_{l}$ is the mass lost at the anode.

The mass lost is also represented as $m_{l}=\rho t_{l}A$, where ρ is the mass density of steel (7850 Kg/m^3^), $t_{l}$ is the thickness lost, and *A* is the surface area of the top face of the strip (Width × Length, 50 mm × 10 mm). In terms of the instantaneous thickness (*t*) and initial thickness of the strip ($t_{i}$), the thickness lost is expressed as $t_{l}=t_{i}-t$; then, Equation (13) becomes:

(14) $$\rho WL\left(t_{i}-t\right)=\frac{IT}{F}\frac{M}{z}.$$

The resistance of the strip in terms of its material resistivity ($\rho_{r}$) is given by the following equation:

(15) $$R=\frac{\rho_{r}L}{Wt}.$$

Substituting Equation (15) into Equation (14) yields the following result for the estimated time *T* to reach a resistance value *R*:

(16) $$T=\frac{\left(t_{i}WR-\rho_{r}L\right)zF\rho L}{RMI}.$$

**Figure 5:**
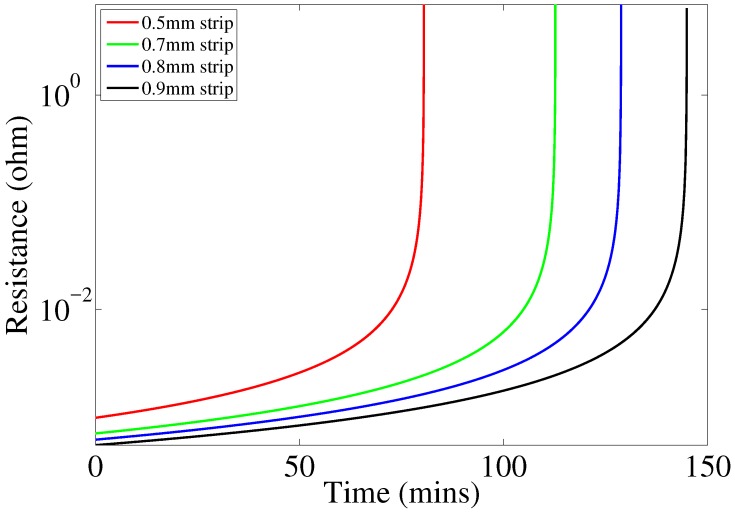
Resistance of sensing strips vs. experiment time.

After plotting the change in resistance of the four strips over time at the constant corrosion rate that corresponds to 1 A current in the corrosion cell, it was observed that resistance changes very slowly just prior to their failure. Therefore, we can model the sensing strips as resistors with constant values of resistance before they fully corrode. Figure 5 shows how the resistance changes with time for all of the four strips.

### 3.2. Experimental Results with Variable Resistors

Initially, in the uncorroded state, the resistance of all strips is very low. Therefore, 10 Ω resistors were initially connected to each LED in the sensor circuit to represent the sensing strips. A variable resistor was then connected in place of the 0.5 mm strip in series with its corresponding LED. Its resistance was gradually increased while keeping the other three resistors constant. The lowest resistance at which the corresponding LED failed to emit light was recorded. The variable resistor was then replaced by a 1 MΩ resistor to indicate an open circuit for the first LED (failure of the 0.5 mm strip).

The same procedure was repeated for other circuit branches representing the 0.7 mm, 0.8 mm and 0.9 mm strips using the same variable resistor to ensure consistency. The resistance value at which the LED fails to emit light was recorded and is shown in Table 1. Using Equation (16), one can also estimate the time necessary to reach the simulated failure resistance values. The obtained values are also shown in Table 1.

**Figure 6:**
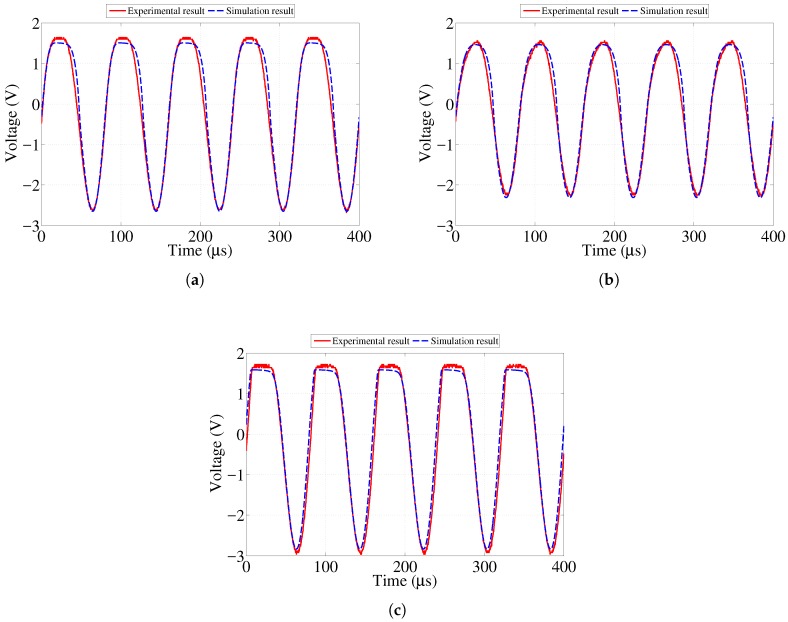
Simulation and experimental voltage data across 0.5 mm strip LED showing response at various resistances. (**a**) 3.8 kΩ resistance (at threshold); (**b**) 6.7 kΩ resistance (above threshold); (**c**) 0.6 kΩ resistance (below threshold).

**Figure 7:**
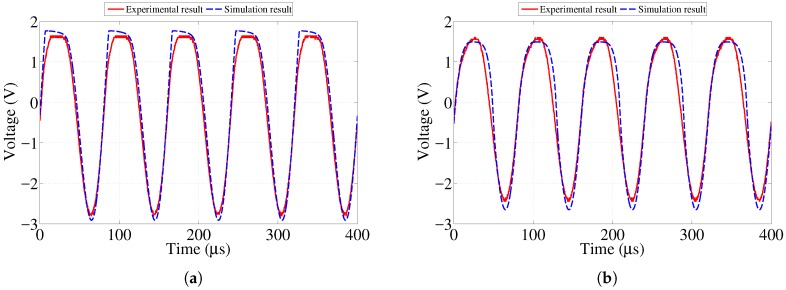

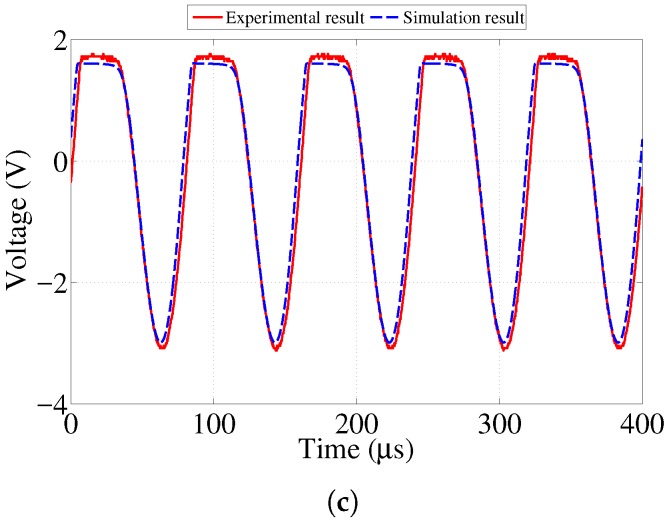
Simulation and experimental voltage data across 0.8 mm strip LED showing response at various resistances. (**a**) 3.8 kΩ resistance (at threshold); (**b**) 6.4 kΩ resistance (above threshold); (**c**) 0.6 kΩ resistance (below threshold).

**Figure 8:**
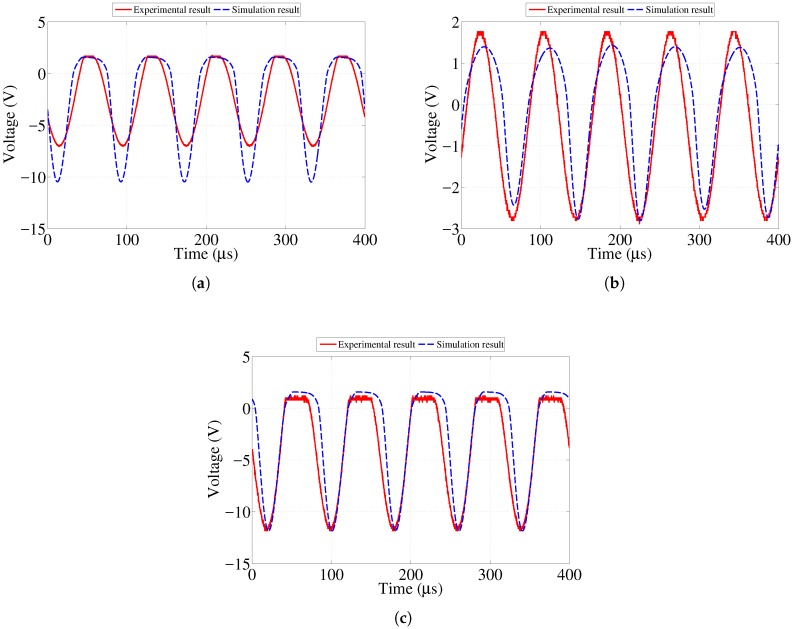
Simulation and experimental voltage data across 0.9 mm strip LED showing response at various resistances. (**a**) 100 kΩ resistance (just above threshold); (**b**) 200 kΩ resistance (far above threshold); (**c**) 32.5 kΩ resistance (below threshold).

Figure 6, Figure 7 and Figure 8 show the numerical and experimental data representing the voltage across the corresponding LEDs obtained at, above, and below the resistance values presented in Table 1. One can observe that the top half of the voltage waveform is sharply clipped at resistance values below the threshold listed in Table 1. This represents the fact that the LED operates in conductive mode and emits light visible to the naked eye. Once the resistance of the sensing strip becomes high enough, the voltage across the LED approaches its threshold level so that it stops conducting and emitting visible light. Note that, in Figure 8a, results are presented for 100 kΩ case, which is close to the threshold value of 83.5 kΩ value reported in Table 1. Although the voltage waveform clipping started to disappear at 83.5 kΩ, it was hardly distinguishable from 32.5 kΩ case shown in Figure 8c. In order to clearly illustrate the ongoing change, it was decided to run and present the case for 100 kΩ value.

It is difficult to predict the behavior of the 0.9 mm strip after failure of all other strips. As the 0.5 mm, 0.7 mm and 0.8 mm strips fail, they create open circuit branches that result in the preferential flow of current through the 0.9 mm strip. Another possible reason behind the discrepancy observed between simulation and experimental results is due to slight differences in the characteristics of LEDs used that might have affected their performance.

## 4. Corrosion Test Experiment

Following good agreement between the model and the data measured from the sensor circuit, an accelerated corrosion test was designed and constructed to test the sensor design under more realistic conditions and demonstrate its ability to visually indicate the level of corrosion by energizing the LEDs associated with sacrificial metal strips.

### 4.1. Accelerated Corrosion Test

Different standard test methods have been studied that help maintain a corrosive environment to accelerate corrosion in the test specimen. However, since accelerated corrosion tests are performed under controlled conditions with a limited number of variables, they often fail to be an exact duplicate of the real world environment. For that reason, comparison standards should be used to compensate for this. Some of the ASTM (American Society for Testing and Materials) standard practices for conducting corrosion tests are discussed in Refs. [26,27,28]. In this work, an electrochemical cell was employed to accelerate corrosion in the manufactured array of metal strips in order to reduce experimental time.

Four steel strips were manufactured to have the same width and length (10 mm × 50 mm) and thicknesses of 0.5 mm, 0.7 mm, 0.8 mm, and 0.9 mm. The metal strips were assembled in a fixture. The fixture was custom designed for the available strips and 3D printed from ABS (Acrylonitrile Butadiene Styrene) plastic. Nylon bolts and nuts were used to fasten different parts of the fixture in order to exclude the risk of any metallic interference, other than the strips, throughout the experiment. To enable the connection of the metal strips to the sensor circuit, electric wires were soldered to the ends of each strip. High viscosity epoxy adhesive was used to insulate the soldered areas on the strips and prevent galvanic corrosion from occurring at the joints.

### 4.2. Experimental Setup

A testing technique for accelerating corrosion was adopted from Ref. [29]. However, the sensor concept presented in this work does not rely on any pre-stressing of the sensing element, and the fixture used here ensures stress-free mounting of the strips. An electrochemical cell was prepared in which the metal strips were used as the anode and graphite electrodes as cathodes because graphite has a higher electrochemical potential than mild steel. The electrolyte was made of distilled water with 3.5% fine sea salt content (standard sea water salinity content). A power supply was connected to the electrodes to supply them with current, which allows for initiating and accelerating the chemical corrosion process. Two Fluke 289 True-RMS multimeters (Fluke Corporation, Everett, WA, USA) were used to log both the current and voltage output of the power supply throughout the experiment. The current output of the power supply remained constant throughout the experiment at about 4 A (maintaining 1 A per strip when possible), while the voltage output varied from 8 V to 10 V. The intention of this test is not in replicating the corrosivity of real outdoor environment, but to speed up the corrosion process to test our sensor design concept.

**Figure 9:**
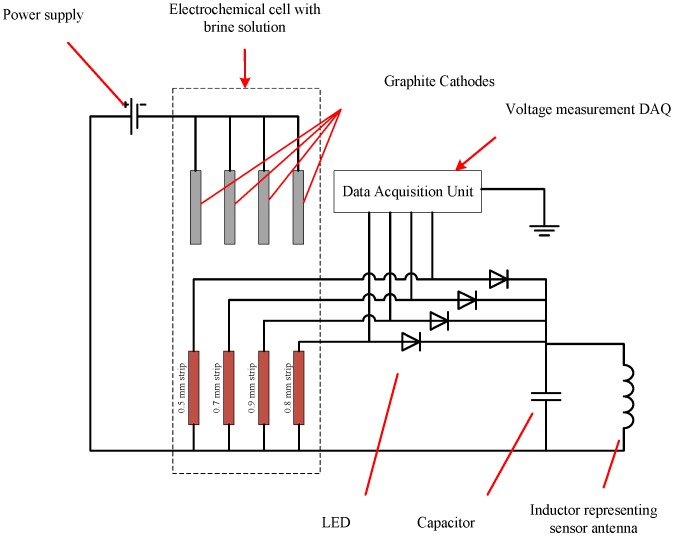
Schematic diagram of the corrosion test experimental setup.

Throughout the experiment, the metal strips were connected to the sensor circuit, which was energized wirelessly by the interrogator circuit. The distance between the interrogator and sensor antennas was fixed at 2 mm throughout the experiment using a height gauge. The current passing through each of the strips and the voltage across each LED in the sensor circuit was monitored using a data acquisition module connected to a PC with LabVIEW software (LabVIEW 2013, National Instruments Corporation, Austin, TX, USA). Figure 9 shows a schematic diagram describing the connections made for the four strips used in the experiment. The fixture assembly with the sensing strips was positioned in parallel and at equal distances to the graphite cathodes.

**Figure 10:**
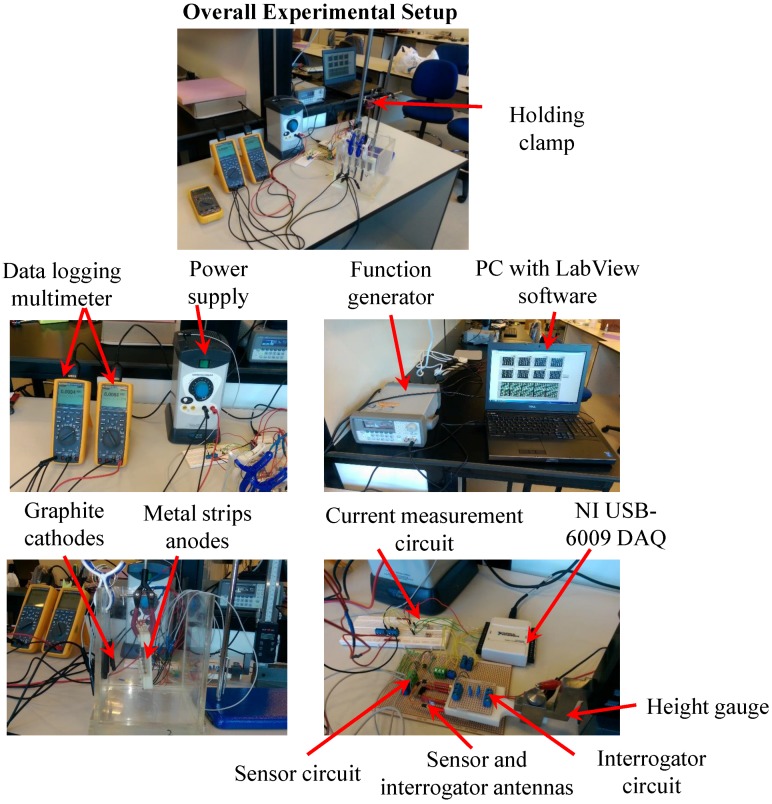
Corrosion test experimental setup.

Figure 10 shows the overall experimental setup prior to switching on the power supply along with detailed pictures of different sections of the setup.

## 5. Results and Discussion

Two sets of results were obtained from the accelerated corrosion test: a visual indication via the LEDs at the time of failure of each metal strip provided an indication of its status, and the LEDs’ voltage response data was acquired via commercial Data Acquisition (DAQ) modules.

### 5.1. LED Status and Visual Failure Indication

Table 2 shows the time of failure of each strip, its condition and the initial current measured through each strip from the power supply. As expected, the thinnest 0.5 mm strip failed first. It can be noticed that the 0.8 mm and 0.7 mm strips failed at approximately the same time, which disagrees with the expectations from theoretical estimation. This occurred due to the uneven distribution of current flowing through the strips, causing more current to flow through the 0.8 mm strip than 0.7 mm strip. A larger value of current in the 0.8 mm sensing strip resulted in a faster corrosion rate. If a thicker strip fails earlier than a thinner one during actual field application, one can conclude that locally much higher corrosion rates may be observed.

Throughout the experiment, bubbles of hydrogen and chlorine gases were observed to form at the cathodes and anodes, respectively, as well as precipitates of iron on the bottom of the electrochemical cell. Final results observed after running the experiment for an hour show that the corrosion process in the electrochemical cell was not uniform, as it was concentrated in the lower section of the strips causing them to fail near their lower ends.

### 5.2. Voltage Response of LEDs throughout Experimental Run

**Figure 11:**
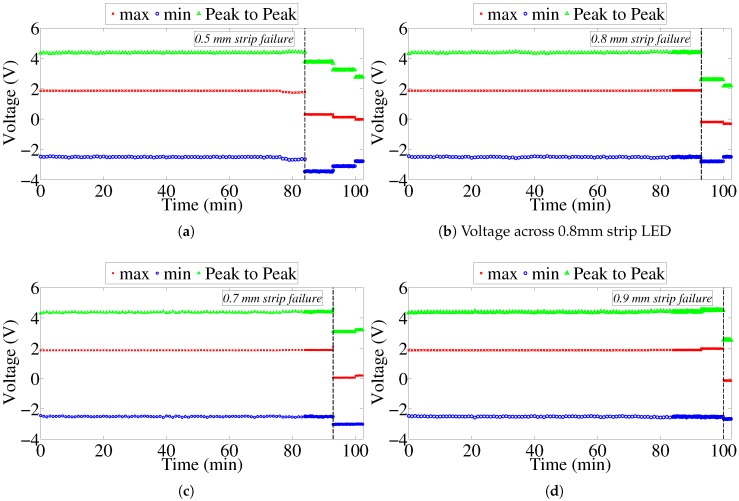
Maximum, minimum and peak-to-peak voltages across different strip LEDs. (**a**) voltage across 0.5 mm strip LED; (**b**) voltage across 0.8 mm strip LED; (**c**) voltage across 0.7 mm strip LED; (**d**) voltage across 0.9 mm strip LED.

By looking at the peak-to-peak voltage across each LED, it can be noticed that the peak-to-peak voltage remained constant throughout the experiment, and, when failure occurred, it decreased suddenly as shown in Figure 11. Moreover, the graphs indicate the time of failure of each strip. The experiment was paused following failure to retrieve the specimens and inspect their conditions. This discontinuity in the experimental time has been removed from all reporting figures to show a continuous experimental run.

**Figure 12:**
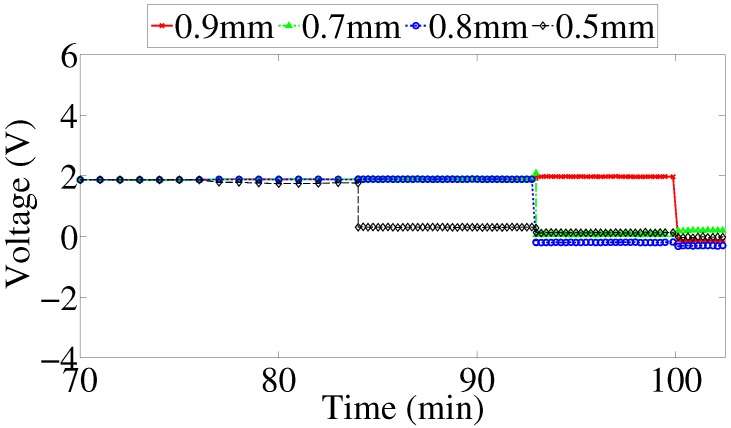
Peak amplitude of voltage across LEDs corresponding to their metal strips.

Furthermore, a close inspection of the peak amplitude of the voltage across the LEDs provides insight into when exactly did failure occur, resulting in switching off the LED, which provides a visual indication of failure. Figure 12 provides a detailed illustration of the peak voltage across the LEDs. Discontinuities in the voltage indicate that the metal strip can no longer conduct the current to the LED.

**Figure 13:**
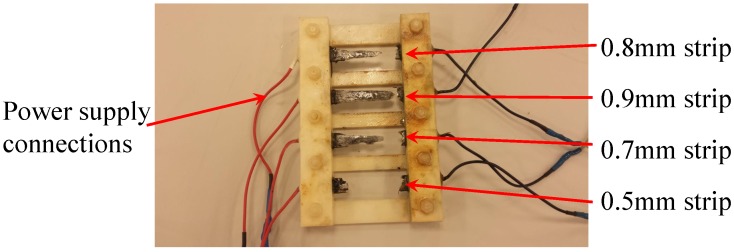
Metal strips at the end of the experiment.

Figure 13 shows the strips at the end of the experiment. It can be observed that even after the failure of the strips, corrosion continued causing further metal loss. The corrosion current continued flowing through the strips even after failure, so the material removal process continued even after the failure of each sensing element, as would occur under natural environmental corrosion conditions.

Overall, results presented here are aligned with the intended operation principle of the proposed sensor and demonstrate its capability of providing a visual indication of its status.

## 6. Conclusions

In this work, we presented a study of the proposed corrosivity sensor that could be suitable for deployment in a hazardous environment, such as that near oil and gas fields or production facilities. The proposed sensor is based on wireless energy transfer and does not need a constant power supply. It consists of two main parts: the sensing array of sacrificial steel strips and the sensor circuit. The sensor provides a visual indication to the operator via LEDs in the circuit regarding the degree of corrosion that took place on the sensing array.

We developed and validated a model that can be used to refine and optimize the sensor design. The developed model will allow for optimizing the operation of the circuit and investigating design and operation of the sensor in alternative frequency bands. This is important because it may allow to reduce the size of components and reduce the overall manufacturing cost. In addition, we performed an accelerated corrosion test to demonstrate the operating principle of the sensor in an actual corrosive environment. Our findings suggest that overall the sensor works as intended; however, its performance can be affected by non-uniformity of corrosion processes occurring on the sensing array. The sensor is only capable of local atmospheric corrosivity measurements, and will help to classify the environment to which the pipe is subjected. It will not be able to detect local differential aeration cells causing the pipeline to corrode, i.e., the sensors may show no corrosion in their locality, while the actual pipeline is corroding.

In some situations, it may be necessary to evaluate corrosivity of environment during a relatively short time period (e.g., during a particular season), while, in other situations, it may be of interest to measure corrosivity over a long period of time (e.g., comparable to the lifetime of a pipeline). The proposed sensor concept and the model presented in this paper allow the development of corrosivity sensors that will have different response times by varying the thickness of metal strips in the sensing array. Sensors with short response times will have to have very thin metal strips and would have to be manufactured using nanofabrication techniques. The sensor circuit needs to be packaged on to a printed circuit board and properly shielded from the environment.

## Figures and Tables

**Table 1:** Estimated time of failure for each strip.

Strip Thickness (mm)	0.5	0.7	0.8	0.9
Threshold resistance (measured) (kΩ)	3.8	3.8	3.8	83.5
Time to failure (calculated) (min)	80	112	129	145

**Table 2:** Time to failure of each strip, initial current measured through each strip and an image of each.

Strip Failed	Time to Failure (min)	Current (A)	Sensor Condition	LEDs Condition
0.5 mm	84	1.19	0.8 mm|0.9 mm|0.7 mm|0.5 mm 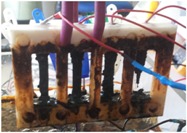	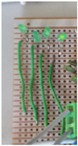
0.8 mm	93	0.94	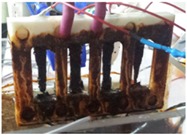	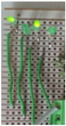
0.7 mm	93	0.76	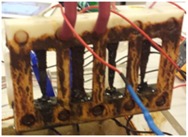	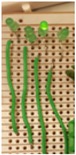
0.9 mm	100	1.08